# Adaptive intrapatient dose escalation of cisplatin in combination with low-dose vp16 in patients with nonsmall cell lung cancer

**DOI:** 10.1038/sj.bjc.6600794

**Published:** 2003-03-18

**Authors:** J H M Schellens, ASTh Planting, N van Zandwijk, J Ma, M Maliepaard, M E L van der Burg, M de Boer-Dennert, E Brouwer, A van der Gaast, M J van den Bent, J Verweij

**Affiliations:** 1 The Netherlands Cancer Institute, Plesmanlaan 121, 1066 CX Amsterdam, The Netherlands; 2 Faculty of Pharmacy, Utrecht University, Utrecht, The Netherlands; 3 Rotterdam Cancer Institute (Dr Daniel den Hoed Kliniek)/University Hospital Rotterdam, PO Box 5201, 3008 AE Rotterdam, The Netherlands

**Keywords:** cisplatin, adaptive intrapatient dose adjustment, platinum DNA adducts, pharmacokinetics, nonsmall cell lung cancer

## Abstract

The objective of this phase II and pharmacologic study was to explore the feasibility, toxicity and activity of adaptive intrapatient dose escalation of cisplatin in a dose-intensive weekly schedule using predefined levels of exposure, with the ultimate aim to improve the antitumour activity of the therapy in patients with nonsmall cell lung cancer (NSCLC). Platinum DNA-adduct levels in peripheral white blood cells during treatment were used as the primary parameter for adaptive dosing. If DNA-adduct levels were not available, the area under the concentration–time curve (AUC) of unbound platinum in plasma was used for dose adaptation. Target levels for DNA-adducts and AUC have been defined in a previously performed pharmacologic study. The feasibility of adaptive dosing was tested in 76 patients with stage IIIB and IV NSCLC, who were planned to receive 6 weekly courses of cisplatin at a starting dose of 70 mg m^−2^, together with daily low oral dose of 50 mg VP16. In total, 37 patients (49%) who were given more than one course received a dose increase varying from 10 to 55%. The majority of patients reached the defined target levels by a dose increase during course two. Relevant grade 2 neurotoxicity was observed in eight (10%) patients and reversible ototoxicity grade 2 in 14 (18%) patients. The strategy of adaptive intrapatient dose adjustment of cisplatin is practically feasible in a research setting even when results for dose adaptation have to be reported within a short time-period of 1 week. The toxicity appeared to be manageable in this cohort of patients. In some patients, exposure after the standard dose was substantially lower than the defined target level and significant dose escalations of more than 50% had to be applied. The response rate (RR) was relatively high: overall 40% (29 out of 72 patients) partial remission (PR), in patients with stage IIIB the RR was 60% (15 out of 25 patients) and with stage IV 30% (14 out of 47 patients). Randomised studies are needed to determine whether the adaptive dosing strategy results in better efficacy than standard dosing.

The prognosis of patients with advanced nonsmall cell lung cancer (NSCLC) remains poor and this stresses the need to develop novel treatment strategies. Cisplatin is one of the most frequently applied agents in the treatment of advanced NSCLC. Treatment with cisplatin-based chemotherapy has a small but statistically significant positive influence on survival (Ruckdeschel, 1990; Souquet *et al*, 1995; Non-small Cell Lung Cancer Collaborative Group, 1995; Van Zandwijk and Giaccone, 1996). Trials comparing supportive care and supportive care plus cisplatin-based chemotherapy revealed a 27% reduction in the risk of death and an increased 1-year survival of 10% (Non-small Cell Lung Cancer Collaborative Group, 1995). Another meta-analysis showed that the response rate of cisplatin in combination with VP16, or various other anticancer agents, was 34% in stage IIIB and only 22% in stage IV disease (Ramanathan *et al*, 1997). Cisplatin is also one of the most active agents in the treatment of advanced cervical cancer, mesothelioma, and head and neck (H/N) cancer. Cisplatin is used in the standard treatment for metastatic ovarian cancer and testicular cancer (Loehrer and Einhorn, 1984; Krarup-Hansen and Hanse, 1991; Kaye *et al*, 1992; Bajorin *et al*, 1993). In all these tumour types, including NSCLC, further improvement of therapy with cisplatin has been extensively investigated. Cisplatin has been used in combinations with other different chemotherapeutic agents, such as DNA-alkylators, topoisomerase II inhibitors, vinorelbine and gemcitabine (Fukuoka *et al*, 1992; Liu, 1993; Sculier *et al*, 1994; Gridelli *et al*, 1996; Wozniak *et al*, 1998; Lippe *et al*, 1999). Besides evaluation of different combinations of cisplatin, important other approaches have focused on increasing the dose and/or the dose intensity of the drug using higher doses per course (Klastersky *et al*, 1986; Gandara *et al*, 1993), or shortening the treatment interval (Planting *et al*, 1993, 1994, 1995a, 1995b, 1996a, 1996b, 1997, 1999). Cisplatin when applied as a single agent at a 3- or 4-weekly schedule and a dose of 100 mg m^−2^ has a low activity in advanced NSCLC and the overall response rate (RR) varies from 12 to 15% (Gandara *et al*, 1993; Wozniak *et al*, 1999). In several tumour types, a significant relation has been suggested between the dose intensity of cisplatin and the likelihood of response and response duration (Ozols and Young, 1984; Gandara *et al*, 1989; Kaye *et al*, 1992). For NSCLC results have been less clear (Klastersky *et al*, 1986; Gandara *et al*, 1993; Gralla *et al*, 1998).

The highest dose intensity reached in these studies was 41 mg m^−2^ week^−1^ (Gandara *et al*, 1993). The dose per course of cisplatin is limited because of the induction of acute intolerable side effects, in particular acute gastrointestinal (GI) toxicity, ototoxicity and renal dysfunction. In most patients, GI toxicity and renal toxicity can adequately be prevented by use of 5HT3 blockers in combination with dexamethasone as antiemetics and by rigorous pre- and posthydration. Inspite of these measures, the dose per course can reasonably not be pushed significantly higher than approximately 100 mg m^−2^. Since cisplatin as a single agent has little myelosuppressive effects when applied at standard doses a realistic option is to shorten the treatment interval. This has extensively been explored in several phase I and II studies. In these studies, cisplatin has been applied in weekly schedules as a single agent as well as in combination with VP16 and recently also paclitaxel (Planting *et al*, 1993, 1994, 1995a, 1995b, 1996a, 1996b, 1997, 1999). In the weekly schedule of cisplatin, VP16 was added at a daily low oral dose, because the two drugs appear to act at least additively (Tsai *et al*, 1989; Wampler *et al*, 1992) and maybe even synergistically, which has been established in preclinical models (Kanzawa *et al*, 1997). In the phase II studies, cisplatin treatment was found active when used at a dose of 70 mg m^−2^ in six courses during 7 weeks (i.e. week 1,2,3 and 5,6,7) in combination with VP16 in advanced cervical cancer, pleural mesothelioma, melanoma and NSCLC (Planting *et al*, 1997, 1999).

In a pharmocokinetic–dynamic study in 29 patients who received weekly cisplatin plus daily low-dose VP16, a significant correlation was found between the area under the unbound plasma concentration–time curve (AUC) of cisplatin (measured as platinum by atomic spectroscopy (AAS)) and the likelihood of tumour response (Schellens *et al*, 1996). In addition, a highly significant difference was found between DNA-adduct levels of platinum, as measured in peripheral white blood cells (WBC) by AAS in responders (*n*=10) and non-responders (*n*=19). The adduct level in responders was 55% higher compared with nonresponders at 1 h after the end of the 3-h infusion of cisplatin. Also at later time points, the responders had significantly higher WBC DNA-adduct levels than nonresponders. There was also a highly significant correlation (*P*<0.001) and linear relation between the AUC of cisplatin and the DNA-adduct levels in WBC indicating that variation in the adduct levels is largely determined by pharmacokinetic variability of cisplatin. In two recent studies, the feasibility of intrapatient dose adjustment has been evaluated (manuscript submitted). This has resulted in the current study to adapt doses of cisplatin during treatment using as a target the mean value of the platinum DNA-adduct levels in WBC and AUC of unbound platinum as observed during course one in the responding patients of the previous pharmacologic study (Schellens *et al*, 1996).

## METHODS

### Selection of patients

Patients were eligible if they had histologically confirmed stage IIIB or IV NSCLC, they were older than 18 and younger than 80 years, had a life expectancy of at least 3 months, if they had measurable disease according to WHO criteria (WHO, 1979), a WHO performance score of 2 or better, adequate bone marrow function (WBC >3.0 × 10^9^ l^−1^, platelets >100 × 10^9^ l^−1^), adequate liver (serum bilirubin <25 *μ*mol l^−1^, serum albumine 25 g l^−1^) and renal function (serum creatinine <140 *μ*mol l^−1^, or creatinine clearance >45 ml min^−1^). They were not eligible if they had received radiotherapy on the indicator lesion, or when any radiotherapy was given within 4 weeks prior to start of the study. New measurable metastases in previously irradiated areas were accepted as indicator lesions. Patients were also ineligible if they had neurologic disease that could cause an increased risk for peripheral or central neurotoxicity, if they had uncontrolled infections, if they were pregnant or were lactating, or if they had known cerebral or leptomeningeal metastases. Previous treatment with cisplatin or carboplatin was not allowed. Patients had to give written informed consent. The study was approved by the local ethics committee.

### Treatment schedule

Cisplatin was administered in six courses during 7 weeks, which was on days 1, 8, 15, 29, 36 and 43. Low-dose VP16 was given as an oral daily dose of 50 mg on days 1–15 and 29–43. Cisplatin was administered in 250 ml of 3% NaCl as a continuous i.v. infusion of 3 h. Patients were prehydrated with 0.75 l dextrose/saline plus 20 mmol KCl and 2 g MgSO_4_ administered in 3 h prior to the 3-h infusion of cisplatin. After the end of the cisplatin infusion, patients received posthydration with 2 l of dextrose/saline plus 40 mmol KCl and 4 g. MgSO_4_ administered over 14 h.

### Blood sampling for pharmacokinetic and platinum DNA-adduct measurements

During each of the first three courses, blood samples were to be taken at 0, 1, 2, 3, 3.5, 4, 5, 6 and 21 h after start of the infusion. The volume of each sample was 4 ml except at time points 0, 4 and 21 h where a volume of 16 ml was collected. Samples at these three time points were also used for the collection of WBC and measurement of platinum DNA-adduct levels, according to a previously validated quantitative assay (Ma *et al*, 1995).

During each of the last three courses only three blood samples were taken of 16 ml at time points 0, 4 and 21 h after start of the infusion of cisplatin.

### Urine collection

During the first three courses 24 h urine was collected in two portions for measurement of the platinum excretion.

### Dose-individualisation

**Figure 1:**
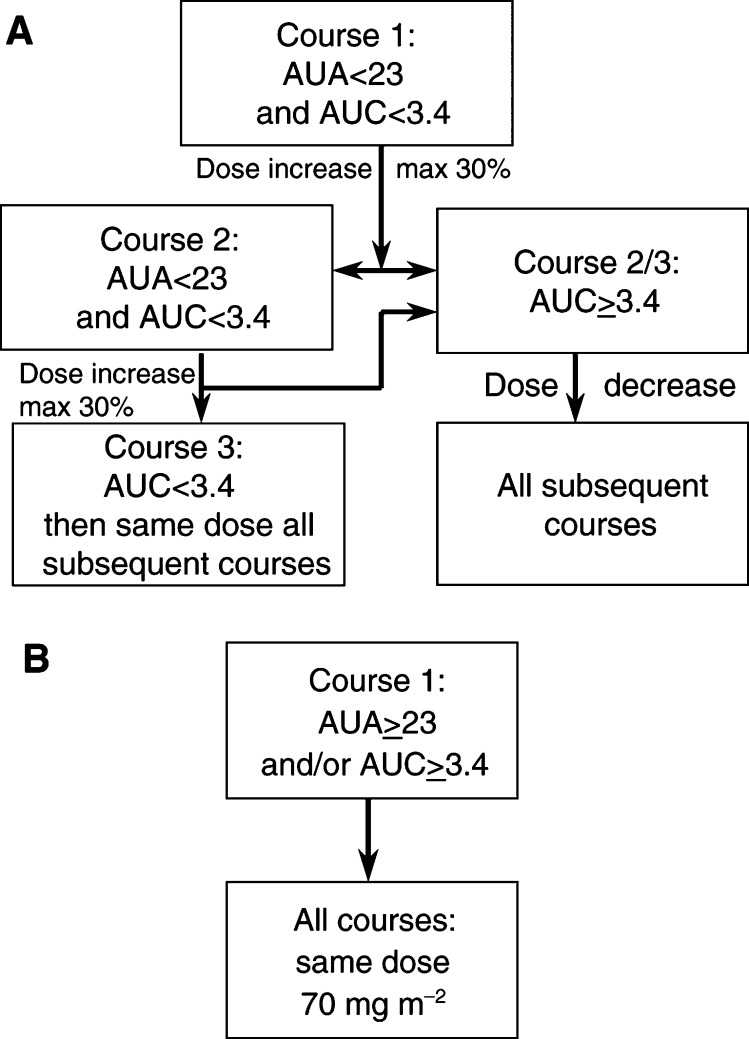
Algorithm for dose adaptation of cisplatin: (**A**) if the observed AUA during course one is below the target of 23 (pg Pt h *μ*g^−1^ DNA) and the AUC is below the safety limit of 3.4 (*μ*g h ml^−1^). (**B**) if the observed AUA during course one is already higher than the target of 23 (pg Pt h *μ*g^−1^ DNA) and/or the AUC is higher than or equal to the safety limit of 3.4 (*μ*g h ml^−1^). (For further details see Methods section.)

The dose of cisplatin that was to be administered during the second course depended on the pharmacokinetic measurements of cisplatin in plasma and the DNA-adducts in WBC. The secondary target was the AUC of unbound platinum in plasma. The algorithm for dose adaptation is summarised in Figure 1

. The starting dose in all patients was 70 mg m^−2^ according to the previously performed phase II study (Planting, 1996). As a basis for dose-individualisation, the pharmacologic data were used of a large pharmacokinetic–pharmacodynamic study in 45 patients who received cisplatin at a dose of 70 or 80 mg m^−2^ (Schellens *et al*, 1996). In that study, the mean AUA (area under the DNA-adduct concentration–time curve) in the group of responders to cisplatin therapy was rounded off to 23 (mean 22.6, range 11.5–32.1 (pg PT h *μ*g^−1^ DNA)). In the nonresponders, the mean AUA was rounded off to 14 (mean 13.7, range 7.4 – 21.3 (pg Pt h *μ*g^−1^ DNA)). The AUA value of 23, the mean value observed in the responders, was taken as the target value for the current dose-individualisation study.

If, in the current study, the observed AUA during course one was below this defined target value of 23, the patient received a subsequent dose increase in order to achieve an AUA value of 23 during the second course. If the observed AUA during the second course was found to be below 23 again, then a second dose increase was applied. The maximum allowable dose increase was set at 30% per course for safety reasons. If the observed AUA during the first course was found to be higher than 23, then no dose reduction was applied, and the patient continued treatment at the starting dose of 70 mg m^−2^ during all courses, since the safety of this dose had previously been shown.

In the previous pharmacologic study, the mean AUC in the responders was 3.0 (range 2.30 – 3.82 (*μ*g h ml)^−1^) and the AUC in nonresponders was 2.2 (range 1.10 – 3.16 (*μ*g h ml)^−1^). If, in the current study, for any reason the AUA could not be determined then a target AUC value of 3.0 was used for adaptive dosing. Hence, if the observed AUC was below 3.0 during course one a dose increase was applied during course two and if the observed AUC during course two was still below 3.0 then a second dose increase was applied with a maximum increase per course of 30%, exactly according to the approach as outlined for the AUA. If the observed AUC during course 1 was higher than the defined target of 3.0, then no dose reduction was applied.

In the previous pharmacologic study also a significant relation was found between the AUC as well as the AUA and the toxicity, in particular the thrombocytopenia. The AUC, but not AUA, was also significantly correlated with the neurotoxicity (in particular, the log vibration perception threshold). For this reason, a maximum value of the AUC was accepted, which was arbitrarily set at 3.4 (the mean value in the responders plus one standard deviation, as observed in the pharmacologic study). If, for example, a patient needed a dose increase, because the observed AUA was below the defined target, but the AUC after the planned dose increase was expected to exceed the safety limit of 3.4, then no or a lower than planned dose increase was applied for safety reasons.

### The dose and schedule of VP16 were not changed during the study

After the end of the planned six courses of cisplatin, patients who showed a tumour response and no unacceptable toxicity would in principle be treated with 21-day courses of daily oral 50 mg VP16 b.i.d., courses to be repeated on day 28 according to the schedule of the previously performed phase II study in NSCLC (Planting, 1996). However, continuation of treatment was left to the discretion of the responsible physician.

### Follow-up studies

Prior to start and every week during treatment a physical examination was performed and the toxicity score (according to the Common Toxicity Criteria (CTC)) and WHO performance score were determined. In addition, haematologic parameters (haemoglobin, leukocyte, granulocyte and platelet counts), serum chemistry (liver (ASAT, ALAT, gamma-GT, LDH, alkaline, phosphatase, bilirubin) and renal function (serum creatinine and measured creatinine clearance), serum albumin and Na, K, Mg, Ca) were determined weekly.

Neurologic examination (including vibrametry) and audiometry were performed prior to start and 2 weeks and 3 and 6 months after the end of cisplatin treatment.

### Pharmacokinetic and pharmacodynamic calculations

The AUC of cisplatin was determined using the noncompartmental trapezoidal method. Cisplatin was measured in plasma as platinum by AAS according to a validated method (Ma *et al*, 1996). The elimination rate constant *k* (h^-1^) was determined using the time points at 4, 5, 6 h after start of the infusion. Curves were extrapolated to infinity by using *C(t)/k*, where *C(t)* is the plasma concentration at the latest time point ‘*t*’ (mostly 6 h after start of infusion). The terminal half-life was calculated by ln 2/*k* (h). The total plasma clearance (CL) of unbound platinum was calculated by Dose/AUC (ml min^−1^).

The area under the DNA-adduct–time (AUA) was determined up to the last measured time point at 21 h after start of the infusion (i.e. by using the time points 0, 4 and 21 h) by applying the trapezoidal method. The parameter AUA has previously been defined (Schellens *et al*, 1996).

Urinary platinum excretion was used to calculate the renal clearance of unbound platinum during the first 24 h after start of treatment.

## TOXICITY AND RETREATMENT

At any subsequent cycle leukocyte counts had to be ⩾2.0 × 10^9^ l^−1^ and platelets ⩾2.0 × 10^9^ l^−1^ 100 × 10^9^ l^−1^. Patients were to be taken off study in case of treatment delay because of drug-related toxicity for more than 2 weeks, and any irreversible ⩾grade 2 nonhaematologic toxicity (in particular neuro-, nephro- and ototoxicity), excluding untreated nausea, vomiting and alopecia. In case patients were taken off study, further treatment was left to the discretion of the responsible physician.

### End points of the study

The clinical and pharmacologic end points of the study were the RR in stage IIIB and IV NSCLC and the toxicity of the treatment and the feasibility of adaptive intrapatient dose adjustment to achieve the desired exposure to cisplatin. The exposure to cisplatin was defined by DNA adducts in WBC and AUC of unbound platinum in plasma. Patients were considered evaluable for response if they had received a minimum of three cycles of cisplatin.

### Statistical analysis

No formal ‘*a priori*’ statistical design was chosen. The study was performed in a relatively large patient population to enable adequate assessment of the feasibility of intrapatient dose escalation. The Pearson correlation coefficients were calculated between dose and exposure parameters where appropriate. The Spearman rank correlation coefficients were calculated between exposure parameters and toxicity scores. *P*<0.05 was defined as statistically significant.

## RESULTS

In total, 76 patients were entered into the study between 1995 and 1999. Study evaluation was complete in 2001. The main characteristics are given in Table 1

**Table 1:** Patient characteristics

Total entered	76
Male	49
Female	27
Median age (range)	56 (33–72)
Median WHO performance score (range)	1 (0–2)
*Stage* III B	27
IIIB with pleural effusion	6
IIIB with T4 tumour	7
IIIB with N3 nodes	14
IV	49
Prior chemotherapy	0
Prior radiotherapy	11

. All patients were evaluable for pharmacokinetics and toxicity. Four patients were not evaluable for response, because they did not receive the minimum number of three courses of cisplatin. Of these patients, two had early disease progression and two refused further treatment because of nausea and vomiting after the first and the second course, respectively.

The 76 patients received in total 381 courses, which means that on average five courses were administered per patient. The achieved number of courses is 84% of the planned maximum of six courses. In all, 18 patients did not complete the planned cisplatin courses, for reasons outlined in Table 2

**Table 2:** Reasons for patients not to complete the six planned cisplatin courses (total entered 76)

**No. of cisplatin administrations**	**No. of patients**	**Reason off study**
1	2	Patient refusal
2	2	Patient refusal, early PD
3	5	3*PD, ototoxicity, delay >2 weeks
4	3	3*delay >2 weeks
5	6	3*PD, 3*delay>2 weeks

. In 15 patients, a treatment delay of 1 week was necessary and in 11 patients of 2 weeks, because of slow recovery of leukocytes and/or thrombocytes. Of the 58 patients who received the planned six courses of cisplatin, 39 patients reached a dose intensity of 60 mg m^−2^ week^−1^, 10 patients with a 1 week delay reached a dose intensity of 52.5 mg m^−2^ week^−1^ and 9 with 2 weeks delay a dose intensity of 47 mg m^−2^ week^−1^. The median dose intensity of all patients was 54 mg m^−2^ week^−1^.

### Pharmacokinetic data, DNA-adduct levels and dose adaptations

**Figure 2:**
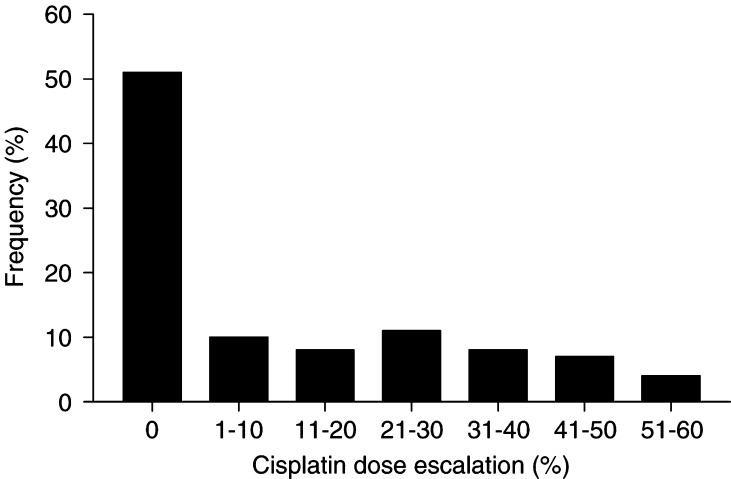
Magnitude of the dose increase of cisplatin expressed as percentage of the starting dose *vs* the percentage of patient (*N*=76).

Of the 75 patients, who received more than one course, in total 37 patients (49%) received a dose increase, because the initial pharmacokinetic parameters were below the defined target level during course one. The magnitude of the dose increases *vs* the percentage of patients is shown in Figure 2

. In individual patients, the dose increase varied from 10 to 55%. The mean dose increase was 28% (Table 2 and Table 3

**Table 3:** Cisplatin dose during courses one and two

	**Course**	**1**	**2**
Cisplatin (mg)	Mean	127	162
	s.d.	15	29
	Range	105–165	105–185
	*N*	76	75

). Of the 37 patients who received a dose increase after the first course, 28 patients reached the target value of DNA adducts during the second course and in nine patients a modest (10–15%) further dose increase was necessary. In four patients, the AUA remained below the target of 23 pg (Pt h *μ*g^−1^ DNA); however, further dose increases were restricted by the high level of the AUC. The AUA and AUC data are given in Table 4

**Table 4:** Area under the DNA-adduct–time curve (AUA) in WBC and AUC of unbound platinum in plasma in 76 patients

	**Course**	**1**	**2**	**3**	**4**	**5**	**6**
AUA (pg Pt h *μ*g^−1^ DNA)	Mean	16.5	22.6	24.9	23.9	26.9	28.9
	s.d.	3.2	4.1	4.6	4.4	5.3	4.8
	Range	10.2–29.0	16.7–39.1	19.5–45.8	16.1–42.1	17.4–42.1	20.6–52.4
	*N*	76	75	65	49	49	31
							
AUC (*μ*g h ml^−1^)	Mean	2.1	2.9	2.7	ND	ND	ND
	s.d.	0.5	0.6	0.4			
	Range	1.1–3.5	2.2–4.1	2.1–4.4			
	*N*	76	75	65			

ND=not determined.

.

The main pharmacokinetic data of cisplatin during course one are given in Table 5

**Table 5:** Total plasma clearance (CL) of unbound platinum (Pt), urinary excretion (0–24 h after start of infusion), renal clearance, and terminal half-life (*t*_1/2_) of Pt in plasma during course one

	**CL unbound Pt (ml min^−1^)**	**Urinary Pt excretion (% dose)**	**Renal CL Pt (ml min^−1^)**	***t*_1/2_ (h)**
Mean	583	29	163	0.47
s.d.	152	6	36	0.16
Range	381–1223	11–38	87–262	0.31–1.06
*N*	76	69	69	76

. Total plasma clearance of unbound platinum was 583±152 ml min^−1^ and renal clearance was 164±36 ml min^−1^. The clearance data obtained during course two and three were of the same order as those of course one.

**Figure 3:**
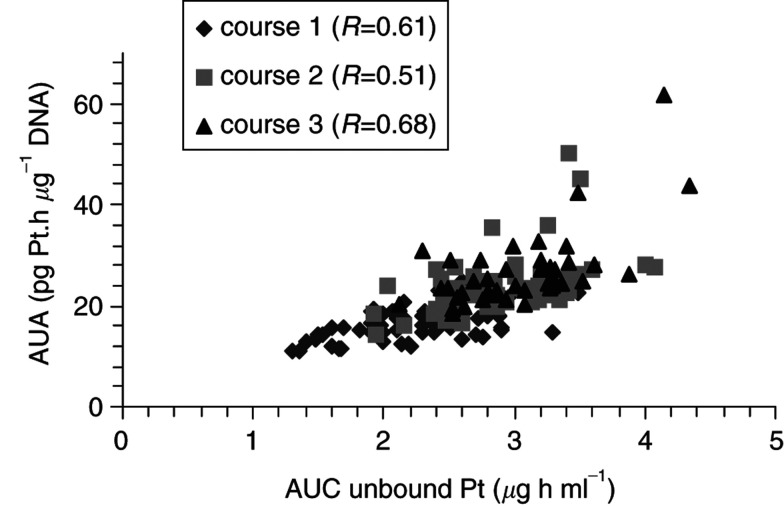
Correlation between the exposure (AUC) to unbound cisplatin during the first three courses and the DNA-adduct levels in WBC (AUA). The correlation coefficients are given (*R*).

The correlation coefficient (*R*) between AUC and AUA during course one was 0.61 (*P*<0.01; *N*=76), during course two 0.51 (*N*=75; *P*<0.01) and during course three 0.68 *N*=65; *P*<0.01) (Figure 3

). The correlation coefficient between dose and AUC during course one was 0.21 (not significant NS; *N*=76) and between dose and AUA 0.17 (NS; *N*=76).

### Tumour response

In total, 72 patients were evaluable for response. None of the patients developed a clinical complete remission (CR). Twenty-nine or 40% of the patients developed a partial remission (PR), 28 (39%) remained stable during the treatment period with cisplatin and 15 (21%) showed progressive disease during or at the end of the planned treatment with cisplatin. The responses per tumour stage are given in Table 6

**Table 6:** Tumour response in 72 evaluable patients with advanced stage IIIB or IV NSCLC

	**Stage IIIB**	**Stage IV**	**All patients**
No. of patients	25	47	72
CR	—	—	—
PR	15 (60%)	13 (30%)	29 (40%)
s.d.	10 (40%)	18 (38%)	28 (39%)
PD	—	15 (32%)	15 (21%)

. The median response duration in stage IIIB was 34 weeks (range 20–54 weeks) and in stage IV 26 weeks (range 14–38 weeks). All patients who developed a PR continued with oral VP16 as single agent. Most patients needed an interval of 2–3 weeks instead of the planned 1 week to recover from grade 1–2 myelosuppression after the end of cisplatin treatment. The median number of courses of VP16 was 3 (range 1–7).

### Toxicity

The main toxicities observed are given in Table 7

**Table 7:** Main CTC graded toxicities in 76 patients that are probably or definitely related to cisplatin therapy. Toxicities are scored as worst grade per patient

**Toxicity**	**Grade 1 *N* (%)**	**Grade 2 *N* (%)**	**Grade 3 *N* (%)**	**Grade 4 *N* (%)**
Leucocytopenia	18 (24%)	24 (32%)	13 (17%)	3 (4%)
Neutropenia	15 (20%)	29 (38%)	16 (21%)	6 (8%)
Thrombocytopenia	21 (28%)	23 (30%)	11 (14%)	2 (3%)
Anaemia	26 (34%)	42 (55%)	0	0
Neurotoxicity	55 (72%)	8 (10%)	0	0
Ototoxicity	29 (38%)	14 (18%)	0	0
Nephrotoxicity	11 (14%)	0	0	0
Alopecia	27 (36%)	16 (21%)	—	—
Nausea	48 (63%)	10 (13%)	4 (6%)	—
Vomiting	27 (36%)	2 (3%)	2 (3%)	0
Diarrhoea	9 (12%)	1 (1%)	0	0
Anorexia	38 (50%)	7 (9%)	0	0
Fatigue	37 (49%)	9 (12%)	0	0

, scored as worst toxicity per patient. The combination of dose intensive weekly cisplatin plus daily low-dose VP16 induced significant myelosuppression with leuko- and granulocytopenia, which necessitated dose delays of 1–2 weeks in 18 patients. Two patients went off study after three courses, four after four courses and three after five courses, because recovery of granulocytes took more than 2 weeks. Mild to moderate anaemia was the most frequent observation and the majority of patients (41 out of 76) needed one or more red blood cell transfusions during treatment. Anaemia developed gradually mostly after three courses. Mild to moderate ototoxicity was observed; however, it did not lead to manifest hearing loss. Most of the reported toxicities started to develop after two to three courses

Also, mild to moderate nausea (grade 1–2) and vomiting (grade 1–2) occurred frequently. Reversible grade 1 nephrotoxicity developed in 14% of the patients and was therefore a relatively infrequent toxicity. Neurotoxicity consisted of mild to moderate paraesthesias, and sensory neuropathy, a common pattern associated with intensive cisplatin therapy, which developed in the majority of patients (72%).

No significant relations were found between exposure parameters (dose, AUC, AUA) and toxicity scores, including neurotoxicity parameters.

## DISCUSSION

Assessment of the optimal treatment of advanced NSCLC has been subject of numerous clinical trials. Cisplatin single-agent therapy is clearly inferior to combination therapy with active agents, including VP16, paclitaxel and gemcitabine. The combination with VP16 has been extensively investigated and the RR in advanced disease averages 30% (Klastersky, 1986). Prospective trials aimed to improve the RR, time to progression (TTP) and survival have focused on dose intensification of cisplatin in combination with VP16. Gralla *et al* (1981) reported that cisplatin 120 *vs* 60 mg m^−2^ plus vindesine every 4 weeks resulted in an RR of 43%, equal in both arms, but the TTP was 12 *vs* 5.5 months and the survival was more than double favouring the high-dose arm. In another randomised study of Gandara *et al* (1989), cisplatin 50 mg m^−2^ on days 1 and 8 *vs* 100 mg m^−2^ on days 1 and 8 in a 4-weekly schedule in stage IV NSCLC resulted in a low RR of 12% in the low dose and 14% in the high-dose arm. In combination with mitomycin C, which was the third arm of the study, the RR was 27%. The highest dose intensity of cisplatin reached was 41 mg m^−2^ week^−1^.

Inspite of these high doses per course the RRs of high-dose cisplatin are disappointing. Randomised studies have thus far not shown survival benefit of regimens applying high doses of cisplatin *vs* standard cisplatin containing treatment schedules in NSCLC (Gandara *et al*, 1989; Font *et al*, 1999). In these studies, all patients received either a high or a standard dose of cisplatin. The interval between the administrations of cisplatin was usually 2–3 weeks. Another important approach to intensify therapy with cisplatin is to decrease the treatment interval, thereby increasing the dose intensity. Studies by Planting *et al* have clearly shown that weekly dose-intensive cisplatin at a dose of 70 mg m^−2^ plus low daily dose of 50 mg VP16 is feasible and active (Planting *et al*, 1993, 1994, 1995a, 1995b, 1996a, 1996b, 1997, 1999). Based on preclinical and clinical data supporting the combination of cisplatin and VP16, VP16 was added in an attempt to improve the activity of cisplatin therapy (Tsai *et al*, 1989, Donnadieau *et al*, 1991; Wampler *et al*, 1992; Kanzawa *et al*, 1997). In NSCLC, weekly 70 mg m^−2^ (weeks 1, 2, 3 and 5, 6, 7) of cisplatin plus low dose of VP16 was investigated in stage IIIA, IIIB and IV patients (Planting, 1996). In the 17 patients with stage IIIB, of whom 13 completed therapy, the overall RR was 35% (six out of 17 patients). In stage IV disease in that study the overall RR in 29 patients was 31%. The same weekly schedule of cisplatin plus VP16 induced favourable RRs in chemo-naive pleural mesothelioma (Planting *et al*, 1994; 1995b), metastatic melanoma (Planting *et al*, 1996b) and advanced cervical cancer (in preparation), tumour types that are known to be only marginally sensitive for chemotherapy.

In a retrospective pharmacologic study, we have shown that the levels of DNA adducts formed in WBC as well as the AUC of unbound platinum in plasma were highly correlated with the likelihood of tumour response in 29 patients with advanced solid tumours, mostly NSCLC, pleural mesothelioma, cervical cancer and carcinoma of unknown primary site who were treated with weekly cisplatin plus daily low-dose VP16 (Schellens *et al*, 1996). Such positive relation was also established in a cohort of 16 patients with advanced H/N cancer treated with cisplatin as the single agent. This has been the starting point for adaptive intrapatient dose escalation with the aim to improve the RR of cisplatin therapy. Two recent explorative studies in cervix cancer (weekly cisplatin plus low-dose VP16) and H/N cancer (weekly cisplatin as single agent) (submitted) revealed that the procedure of dose adaptations using DNA-adduct levels and/or AUC of cisplatin is feasible in a research setting, even when the turnaround time for reporting of analytical and pharmacokinetic results is as short as 1 week.

In the current phase II and pharmacologic study, the RR of the weekly therapy of cisplatin plus low-dose VP16 was the primary end point, besides assessment of the feasibility in a large prospective study. The RR in stage IIIB was 60% in 25 patients evaluable for response and 30% in 47 patients with stage IV disease. The RR of 60% in 25 patients with stage IIIB is encouraging compared with historical controls applying cisplatin in combination with VP16 at a 3- or 4-weekly schedule and also compared with the outlined weekly schedule of cisplatin plus low dose of VP16 (Planting, 1996). Clearly, prospective randomised studies are necessary to reveal whether the activity, and more important the TTP and survival, can be increased by intrapatient dose escalation of cisplatin. In stage IV disease, the RR of our study was in the same range as reported by Donnadieu *et al* (1991) and Planting (1996). However, the RR in the current study appears to be higher than compared with cisplatin at a high dose as single agent at a 3- or 4-weekly schedule (Gandara *et al*, 1989). Therefore, development of other novel approaches is necessary to improve the treatment outcome in patients with stage IV disease.

The 76 patients received on average 5.0, or 84%, of the planned maximum of six courses. This illustrates that despite the dose increase, the dose intensive schedule is practically feasible in chemo-naive patients in good clinical condition. The achieved median dose intensity of cisplatin of 54 mg m^−2^ week^−1^ is relatively high considering the combination with daily VP16. The median dose intensity of cisplatin in our study is comparable to the dose intensity of weekly cisplatin in combination with radiation in cervix cancer.

Prior to the execution of the study a dosing algorithm was designed allowing maximally 30% dose escalations, which was arbitrarily chosen for safety reasons. In most patients one dose escalation was sufficient. In 49% of the patients, a dose increase of 10–55% was necessary to reach the defined target levels of DNA adducts and/or AUC. The wide range of dose escalations reflects the variability in the pharmacokinetics of cisplatin. In 24% (nine out of 37 patients) of the patients who needed a dose increase, a second increase was necessary after the second course, because the target levels had not been reached after the first dose increase. In five patients, the AUC level became higher than the upper limit of 3.4 during course three, the level which was chosen for safety reasons, although from course two to three no dose escalation was applied. Apparently, this was caused by intrapatient variability in pharmacokinetics of cisplatin. The maximum AUC reached was 4.4 in one patient.

In all patients during course one, the DNA-adduct levels could be determined and dose escalations were based on adduct levels. Theoretically, this parameter may be more of interest than the AUC, because the DNA-adduct formation is considered to be the cytotoxic lesion of cisplatin (Eastman and Schulte, 1988). If the current strategy of intrapatient dose escalation is going to be applied at a wider scale in the future, then the study design should be simplified. The AUA was significantly correlated with the AUC of cisplatin (Schellens *et al*, 1996). This is confirmed in the current study, as demonstrated by the significant correlation between DNA-adduct levels in WBC and the AUC during all investigated three courses. The AUC may be used as the main parameter for dose adaptation. In addition, the number of samples for estimation of the AUC of cisplatin may be reduced. At present, the data of this and other studies are being used to design a limited sampling model in order to further improve the practical application of adaptive dosing for cisplatin.

The main pharmacokinetic parameters of cisplatin, in particular plasma clearance, terminal half-life and renal clearance, are in the same range as previously described (Reece *et al*, 1987, 1989; Schellens *et al*, 1996).

The toxicities of the applied dose-intensive cisplatin therapy plus low-dose VP16 are mainly related to neurotoxicity, ototoxicity, haematologic and GI toxicity. The great majority (80%) of patients developed mild (72%) to moderate (8%) neurotoxicity that presented as paresthesias. Ototoxicity was another frequently observed toxicity and 14% of the patients developed relevant but reversible CTC grade 2 toxicity. It is anticipated that in particular this toxicity precludes further dose intensification of cisplatin. This toxicity was also observed in previous high-dose studies (Gandara *et al*, 1989; Planting *et al*, 1995; Planting, 1996). The majority of the patients also developed anaemia, which frequently necessitated blood transfusions. This was also found in previous studies with the weekly schedule of cisplatin plus low-dose VP16 (Planting *et al*, 1995b). On average, the GI toxicity was manageable. Only two patients discontinued therapy because of subjective unacceptable toxicity (scored as CTC grade 2 nausea and vomiting). Renal toxicity was an unusual finding. Only 14% of the patients developed grade 1 toxicity. This may be because of administration of cisplatin in hypertonic (3%) NaCl, rigorous pre- and posthydration and frequent instruction of the patient. We found no significant relation between the dose intensity of cisplatin and the observed toxicities. We anticipate that the main reason for this lack of relation is the narrow range of the exposures to unbound cisplatin during repeated courses. The coefficient of variation in the AUC of unbound Pt was only 15% during course three. The range of the AUC of unbound Pt was three-fold during course one, which reduced to two-fold during courses two and three. Also, the AUA data showed little variation most likely as a results of dose adaptation

In conclusion, the strategy of intrapatient dose adjustment for cisplatin is practically feasible in a research setting even when a short turnaround time of 1 week is the limit for reporting of results. At the applied initial dose level of 70 mg m^−2^ in total 37 (49%) of the patients needed a dose increase, varying from 10 to 55% to reach predefined exposure levels.

The RR of 60% in stage IIIB NSCLC is encouraging. A randomised study comparing individualised dose *vs* standard dose cisplatin applying the same schedule of administration may unravel whether the adaptive dosing strategy results in improved TTP and survival. The latter end point should be considered the primary objective in such pivotal trial. Prior to the execution of such randomised study, we currently explore schedule-intensive cisplatin in combination with gemcitabine as a basis for adaptive dosing in NSCLC. In addition, combined modality of cisplatin-based chemotherapy and radiation can be considered for stage IIIB NSCLC. In stage IV disease, other novel concepts are needed to improve therapy.
